# Ultrathin Freestanding BiFeO_3_ Membranes Grown by Pulsed Laser Deposition

**DOI:** 10.1002/smll.202511535

**Published:** 2026-08-18

**Authors:** Yifan Zhao, Bixin Yan, Christoph Dräyer, Morgan Trassin, Marta D. Rossell

**Affiliations:** ^1^ Electron Microscopy Center Empa Swiss Federal Laboratories for Materials Science and Technology Dübendorf Switzerland; ^2^ Department of Materials ETH Zurich Zurich Switzerland

**Keywords:** cation diffusion, ferroelectric membranes, pulsed laser deposition, scanning transmission electron microscopy, second harmonic generation

## Abstract

Recent advances in the fabrication of single‐crystalline oxide membranes have opened a promising avenue for the realization of freestanding ferroelectric films with potential applications in the field of flexible electronics, such as sensors, actuators, and energy harvesting devices. A widely used fabrication method is the integration of water‐soluble Sr_3_Al_2_O_6_ (SAO) as a sacrificial layer between the perovskite‐type substrate and the ferroelectric film. Here we report the design of few‐unit‐cell‐thick ferroelectric membranes using pulsed laser deposition. This work reveals the tendency for strontium diffusion from the SAO layer into the BiFeO_3_ (BFO) films during the pulsed deposition, leading to the spontaneous formation of an Aurivillius‐like layered phase. Mitigation of such undesired effects is achieved here by introducing a SrRuO_3_ (SRO) buffer layer between the SAO and BFO layers. This prevents strontium diffusion and enables the fabrication of BFO membranes down to 5‐unit cells with high structural integrity and a consistent polar tetragonal distortion. Our results demonstrate that freestanding ferroelectric films can be fabricated with a degree of precision comparable to that of molecular beam epitaxy. Our strategy can be further extended to other membrane compositions.

## Graphical abstract

Ultrathin freestanding BiFeO_3_ membranes down to five‐unit cells are realized by pulsed laser deposition using a water‐soluble Sr_3_Al_2_O_6_ sacrificial layer. Strontium diffusion is revealed to drive the spontaneous formation of an Aurivillius‐like phase, while ultrathin SrRuO_3_ interlayers suppress interfacial intermixing, preserve polar tetragonality, and enable chemically stable, highly controlled ferroelectric membranes.

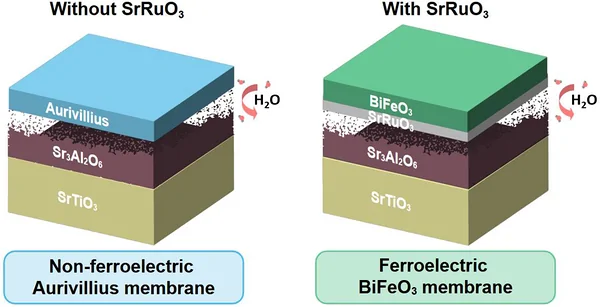

## 1 Introduction

Advances in the fabrication of high‐quality monocrystalline complex oxide membranes—thin films that are not bound to rigid substrates—have enabled the recent development of freestanding ferroelectric membranes and the exploration of their emerging functionalities, including ferroelectricity at the monolayer limit, superelasticity, and exotic polar textures in moiré lattices [1, 2, 3]. More broadly, the ferroelectric membrane design offers an effective strategy to overcome the current limitations of conventional epitaxial thin films and multilayers that constrain device scaling and functionality. On the one hand, the limited availability of suitable substrates restricts heteroepitaxy to a narrow range of material systems with similar crystal structures, lattice orientations, and lattice parameters. Moreover, the elevated growth temperatures typically required for complex oxides hinder the integration of ferroelectrics with established technologies, such as silicon‐based platforms and flexible electronics, thereby limiting their applicability in industry‐relevant devices. On the other hand, the structural constraints imposed by the epitaxial growth severely limit the ability to tailor the mechanical boundary conditions, relegating electrostatics to a secondary role in determining the polarization state [4]. In addition, domain‐wall pining at the substrate/film interface introduces additional energy barriers that must be overcome to achieve efficient ferroelectric switching at reduced dimensions [5].

The fabrication of ultrathin ferroelectric freestanding membranes provides a powerful pathway for realizing high‐performance ferroelectric devices at the nanoscale. By combining reduced dimensionality with the complete release of substrate‐induced constraints, these membranes enable efficient polarization switching, enhanced domain‐wall mobility, and strong strain‐driven responses, all of which are critical for low‐power and highly scalable device operation. In the ultrathin limit, ferroelectric behavior becomes exceptionally sensitive to electrostatic and mechanical boundary conditions, allowing low‐voltage control and access to size‐dependent functionalities that are inaccessible in bulk or substrate‐bound films. Furthermore, the freestanding and mechanically compliant nature of these membranes facilitates integration with flexible and heterogeneous platforms and enables the stacking of layers inspired by 2D van der Waals materials, paving the way for artificial heterostructures and topological architectures with programmable functionalities. Ultrathin ferroelectric membranes therefore represent a versatile materials platform for next‐generation memory, logic, neuromorphic, and nanoelectromechanical devices.

In recent years, several exfoliation and transfer technologies have been developed to produce high‐quality single‐crystal oxide thin films [6, 7]. Wet‐etching of sacrificial buffer layers has emerged as the most reliable method. In this approach, an epitaxial sacrificial layer is grown between the substrate and the ferroelectric layer of interest and subsequently dissolved by selective etching, releasing the freestanding membrane. The water‐soluble Sr_3_Al_2_O_6_ (SAO) [7] sacrificial layer stands out as the most prominent example with its convenient processing and crystalline compatibility with prototypical perovskite ferroelectrics such as BaTiO_3_ (BTO) [8], Pb(Zr_x_Ti_1−x_)O_3_ (PZT) [9] and BiFeO_3_ (BFO) [10]. The complex chemistry and the high tendency for ionic diffusion of the SAO lattice, however, renders the design of ultrathin ferroelectric membranes with high crystalline quality challenging. While low‐energy deposition processes such as molecular beam epitaxy (MBE) have enabled unprecedented observation of ferroelectricity and magnetic orders down to the ultrathin limits in membranes transferred on various substrates, reports of few‐unit‐cell‐thick ferroelectric membranes with high quality based on simpler and more versatile deposition processes such as pulsed laser deposition have remained scarce.

In this work, we establish the design of high‐quality ferroelectric BFO thin film membranes using pulsed laser deposition. We use SAO sacrificial buffers grown epitaxially on SrTiO_3_ (STO) (001) substrates. After water dissolution, the membranes are transferred onto Si wafers and carbon‐coated TEM copper grids for investigation using a combination of optical second harmonic generation (SHG) and scanning transmission electron microscopy (STEM). We reveal that the high‐temperature, high‐energy pulsed laser deposition of the BFO/SAO bilayers results in the Sr diffusion from the SAO sacrificial layer into the BFO films and the spontaneous formation of an Aurivillius‐like Bi_10_Sr_15_Fe_10_O_46_ (BSFO) phase [11]. Most importantly, we show that the insertion of a SrRuO_3_ (SRO) interlayer between BFO and SAO suppresses cation diffusion and enables the fabrication of ultrathin, freestanding BFO membranes. Our results confirm the ability to stabilize a high polar, tetragonal‐like distortion in 5‐unit‐cell‐thick BFO membranes. Hence, our work opens routes for the van der Waals stacking of ultrathin oxide thin films using the versatile pulsed laser deposition process.

## 2 Results and Discussion

### 2.1 Spontaneous Growth of Aurivillius‐Like Bi_10_Sr_15_Fe_10_O_46_ (BSFO) During the BFO Deposition on SAO

We start our investigation of the feasibility of high‐quality ultrathin BFO membranes grown by pulsed laser deposition by testing the macroscopic polar response of a BFO (20 nm)/SAO (170 nm) bilayer grown on a (001)‐oriented SrTiO_3_ (STO) substrate (see Experimental Section) using optical SHG prior to the water‐induced dissolution of SAO. SHG is a nonlinear optical process that describes the frequency doubling of a light wave in non‐centrosymmetric materials and hence, it is a convenient noninvasive tool for the characterization of ferroelectrics [12, 13]. Within the electric dipole approximation the SHG intensity is proportional to the square of the polarization *I*_2ω_∝*P* ^2^ [14, 15, 16]. Figure 1a compares the SHG polarimetry of the BFO/SAO bilayer on STO (labeled hereafter BSFO/SAO/STO and shown in blue) with a reference BFO/SRO/STO multilayer (in green), where SRO stands for SrRuO_3_. In contrast to the clear, characteristic two‐lobe SHG signal from the reference BFO layer on conventional SRO‐buffered STO substrate [14], there is a complete suppression of the SHG signal and hence of the polarization in the BFO layer grown directly on the SAO buffer layer. To further shed light on the lack of polarization in our BSFO/SAO/STO system, we also performed a structural characterization using X‐ray diffraction. Figure 1b shows the θ–2θ diffractograms of BSFO/SAO/STO (in blue), again compared with a reference BFO/SRO/STO system (in green). While a pronounced SAO reflection corresponding to the expected epitaxially enforced orientation is visible, there is no signature of the BFO film. Instead, multiple reflections appear at lower angles, as marked by asterisks in Figure 1c. This suggests the formation of a phase with a larger out‐of‐plane lattice parameter during the early stages of pulsed laser deposition on SAO, likely at the expense of the rhombohedral BFO phase.

**FIGURE 1:**
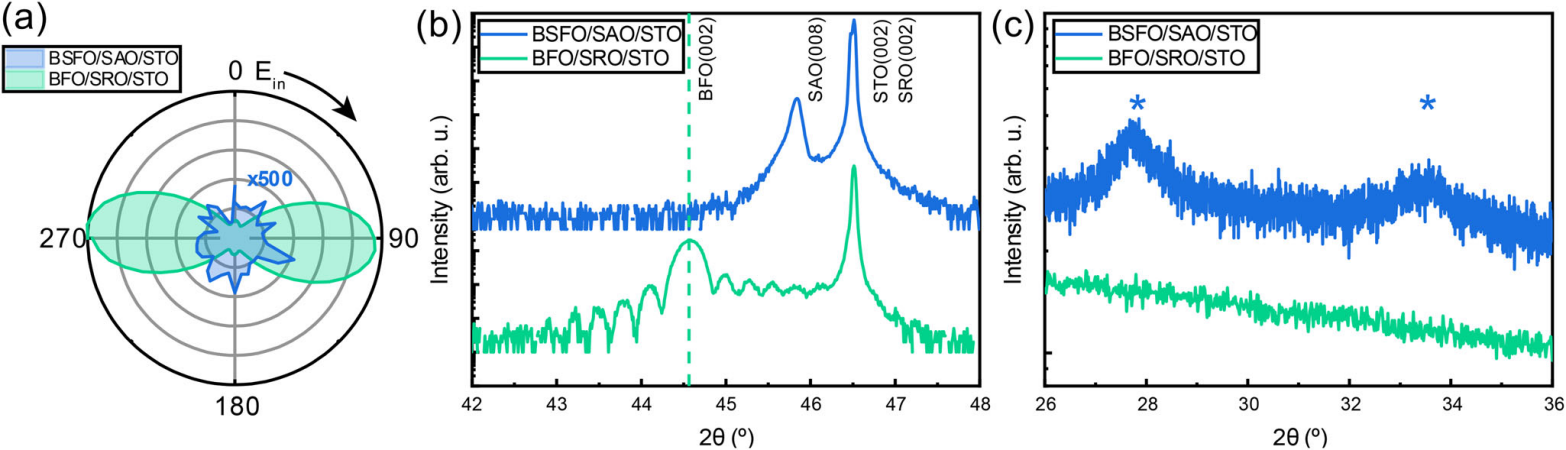
(a) SHG polarizer measurements (see Experimental Section) from the BSFO/SAO/STO (blue) and BFO/SRO/STO reference (green) heterostructures in reflection geometry with a 45° incidence angle for fixed *s*‐polarized SHG light. (b and c) θ–2θ diffractograms of the same samples around the substrate STO (002) reflection and at lower angles. The peaks corresponding to the (10 0 0) and (12 0 0) of the BSFO phase are marked with asterisks. A vertical offset was added to the XRD data for clarity.

The examination of the BSFO/SAO/STO heterostructure by high‐angle annular dark‐field (HAADF) STEM shows that the BFO deposition directly on SAO leads to the formation of a layered compound. Figure 2a shows a low‐magnification and an atomic‐resolution cross‐sectional HAADF‐STEM image of the as‐grown heterostructure. They reveal a layered thin film approximately 15–20 nm thick, composed of intergrowths of two *AB*O_3_ perovskite blocks alternating with fluorite‐type [Bi_2_O_2_]^2+^ layers along the out‐of‐plane direction, reminiscent of Aurivillius structures. The *A*‐type atomic columns in the *AB*O_3_ perovskite blocks have a lower contrast than those in the [Bi_2_O_2_]^2+^ layers. Since the contrast of the HAADF‐STEM images is approximately proportional to the square of the atomic number *Z*, we concluded that Bi (*Z* = 83) is absent in the perovskite blocks, which consist of Sr (*Z* = 38) and Fe (*Z* = 26), as confirmed by energy dispersive X‐ray (EDX) spectroscopy (Figure S1). Thus, it is inferred that the pulsed laser deposition process of BFO on SAO triggers the Sr diffusion into the BFO layer, resulting in the nucleation of an Aurivillius‐like phase with Bi_10_Sr_15_Fe_10_O_46_ (BSFO) composition and SrFeO_3_ perovskite blocks alternating with [Bi_2_O_2_]^2+^ layers [11]. The BSFO phase crystallizes in the orthorhombic *C*222 space group with *a* = 31.696 Å, *b* = 27.245 Å, *c* = 5.462 Å (see Figure 2b). Its crystal structure is centrosymmetric and thus fully correlates with the suppression of the SHG signal and the absence of a net polarization despite the BFO deposition. Hence, an Aurivillius‐like phase with surprisingly minimal defect density grows spontaneously onto the SAO sacrificial layer. This process has been reproduced many times and suggests new design routes for highly anisotropic layered compounds by pulsed laser deposition using simple, perovskite‐based materials, here BFO. We emphasize that, in contrast to previous reports highlighting the need of orthorhombic substrates for the growth of high‐quality Aurivillius thin films [17, 18], phase‐pure layered Aurivillius films can be grown with our approach on most conventional cubic STO substrates.

**FIGURE 2:**
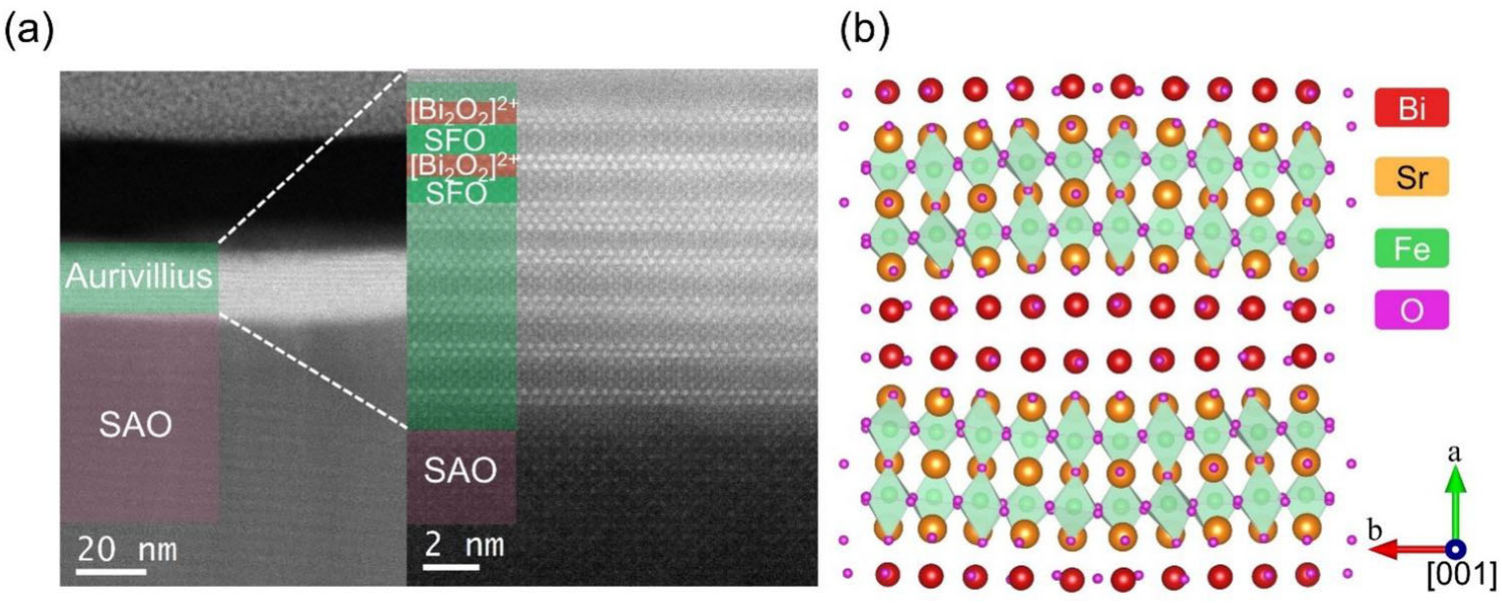
(a) Low‐magnification and atomic‐resolution cross‐sectional HAADF‐STEM images of the as‐grown BSFO/SAO/STO heterostructure imaged along the [015]_o_ direction of the BSFO phase. (b) Schematic [11] of the Bi_10_Sr_15_Fe_10_O_46_ (BSFO) Aurivillius‐like structure with two SrFeO_3_ (SFO) perovskite blocks alternating with [Bi_2_O_2_]^2+^ layers along [001]_o_ [19]. Indexing is based on the BSFO orthorhombic (o) *C*222 space group.

Furthermore, we successfully fabricated Aurivillius‐like BSFO membranes by wet‐etching the SAO sacrificial layer and transferred them onto Si wafers and carbon‐coated TEM grids for cross‐sectional and plan‐view HAADF‐STEM and EDX analyses, as shown in Figures S1 and S2.

### 2.2 Growth of Single‐Crystalline BiFeO_3_ Assisted by a SrRuO_3_ Interlayer

To achieve high‐quality BFO single‐crystalline membranes, we investigated strategies to suppress Sr diffusion in our system. The narrow range of PLD growth parameters for phase‐pure BFO thin films limits the possibility of significantly altering the deposition temperature, laser fluence, or oxygen partial pressure for the BFO layer [20]. We therefore explored the use of interlayers between BFO and SAO as diffusion barriers. Our literature survey identified several studies reporting PLD growth of high‐quality thin films of STO [21, 22, 23], La_0_._7_Sr_0_._3_MnO_3_ (LSMO) [24, 25, 26], LaNiO_3_ [23], and SRO [23, 27] on SAO. Based on these reports, we selected SRO as diffusion barrier to suppress chemical reactivity between the BFO film and SAO, as it supports high‐quality epitaxial growth and is well established as a bottom electrode in ferroelectric switching experiments.

In Figure 3a, we show the polarization‐dependent SHG polarimetry scan of a 35‐nm‐thick BFO layer on STO with an additional 30‐unit‐cell‐thick SRO interlayer (BFO/SRO/SAO/STO). The SRO insertion enables the observation of an SHG signal that fits the monoclinic point group *m* of BFO (Figure S3), indicating that the ferroelectric signature of BFO is recovered on SAO when an SRO interlayer is inserted. We note that the SHG polarimetry pattern differs from that of BFO/SRO/STO (Figure 1a), which we attribute to variations in epitaxial strain affecting the net polarization state and, hence, the SHG response [13, 28]. Reciprocal space mapping (Figure S4) confirms that BFO on SRO/SAO/STO is indeed strained to the SAO lattice rather than to STO, resulting in a distinct compressive strain compared with the BFO/SRO/STO reference (lattice mismatches of ∼0.1% and ∼1.5%, respectively). The θ–2θ diffractogram of the BFO/SRO/SAO/STO heterostructure (in orange, Figure 3b) shows the BFO (002) reflection, previously absent in the BSFO/SAO/STO diffractogram (in blue), proving that SRO buffer layers enable the successful growth of single‐crystalline epitaxially oriented BFO thin films on SAO by pulsed laser deposition. The shift of the BFO peak in the BFO/SRO/SAO/STO heterostructure to higher 2θ values compared to the BFO reference in Figure 1b further reflects the reduced epitaxial compressive strain of the layer.

**FIGURE 3:**
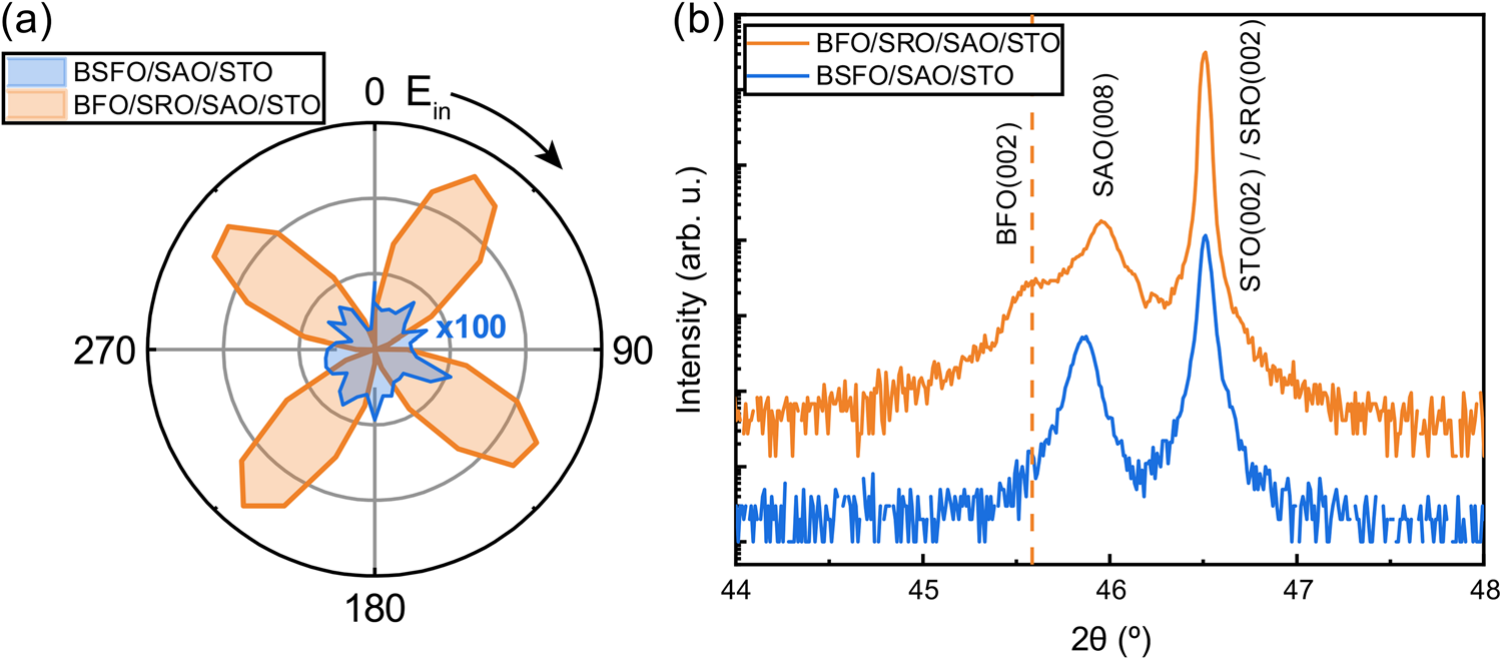
(a) SHG polarizer measurements (see Experimental Section) from the BSFO/SAO/STO (blue) and BFO/SRO/SAO/STO (orange) heterostructures in reflection geometry with a 45° incidence angle for fixed *s*‐polarized SHG light. (b) θ–2θ diffractograms of the same samples around the substrate STO (002) reflection.

The effectiveness of the SRO buffer in suppressing atomic diffusion can be attributed to improved interface compatibility. When BFO is grown on SAO, despite the minimal lattice mismatch of ∼0.1%, cation mobility is enhanced due to the distinct interfacial chemistry and the open tetrahedral framework of SAO. Structurally, SAO is a 3D network of corner‐sharing AlO_4_ tetrahedra with large Sr‐filled cavities, making it far less densely packed than the octahedral frameworks of BFO (FeO_6_) or SRO (RuO_6_). The volatility of Bi during BFO deposition [29], combined with the open SAO framework, likely facilitates Bi diffusion into the underlying layer and concurrent Sr migration from the SAO layer toward the overlying film, locally modifying the SAO interface. In contrast, Sr^2^^+^ is relatively immobile under typical growth conditions and remains largely fixed when SRO is grown on SAO, despite the larger lattice mismatch of ∼‐0.8%. As a result, cation migration occurs for BFO grown directly on SAO but is effectively suppressed by introducing an SRO buffer layer, highlighting the critical role of substrate and buffer selection in controlling interlayer cation diffusion in ultrathin ferroelectric membranes. Substrate‐dependent cation redistribution during PLD has been widely reported in complex oxide heterostructures, particularly in the ultrathin limit where interfacial effects dominate [30, 31].

We now turn to the fabrication of freestanding BFO membranes. As‐grown BFO/SRO/SAO/STO heterostructures were immersed into deionized water and transferred to a Si substrate employing a polydimethylsiloxane (PDMS) stamping process (see Experimental Section). Scanning electron microscopy (SEM) images of the transferred BFO/SRO membranes show crack‐free regions extending over several hundred µm^2^ (Figure S6). Using cross‐sectional HAADF‐STEM imaging, we investigated both the as‐grown BFO/SRO/SAO/STO heterostructure (Figure 4a) and the transferred BFO/SRO membrane (Figure 4b), previously characterized by SHG and XRD (Figure 3). The images reveal the high crystalline quality of the BFO, SRO, and SAO layers and the atomically sharp interfaces between them before and after SAO dissolution. This confirms that the introduction of an SRO buffer layer enables the design of single‐crystalline BFO thin films under exactly the same growth conditions. In addition, EDX analysis of the BFO/SRO/SAO/STO heterostructure demonstrates that the SRO buffer layer effectively prevents the diffusion of Sr into the BFO layer (Figure S5).

**FIGURE 4:**
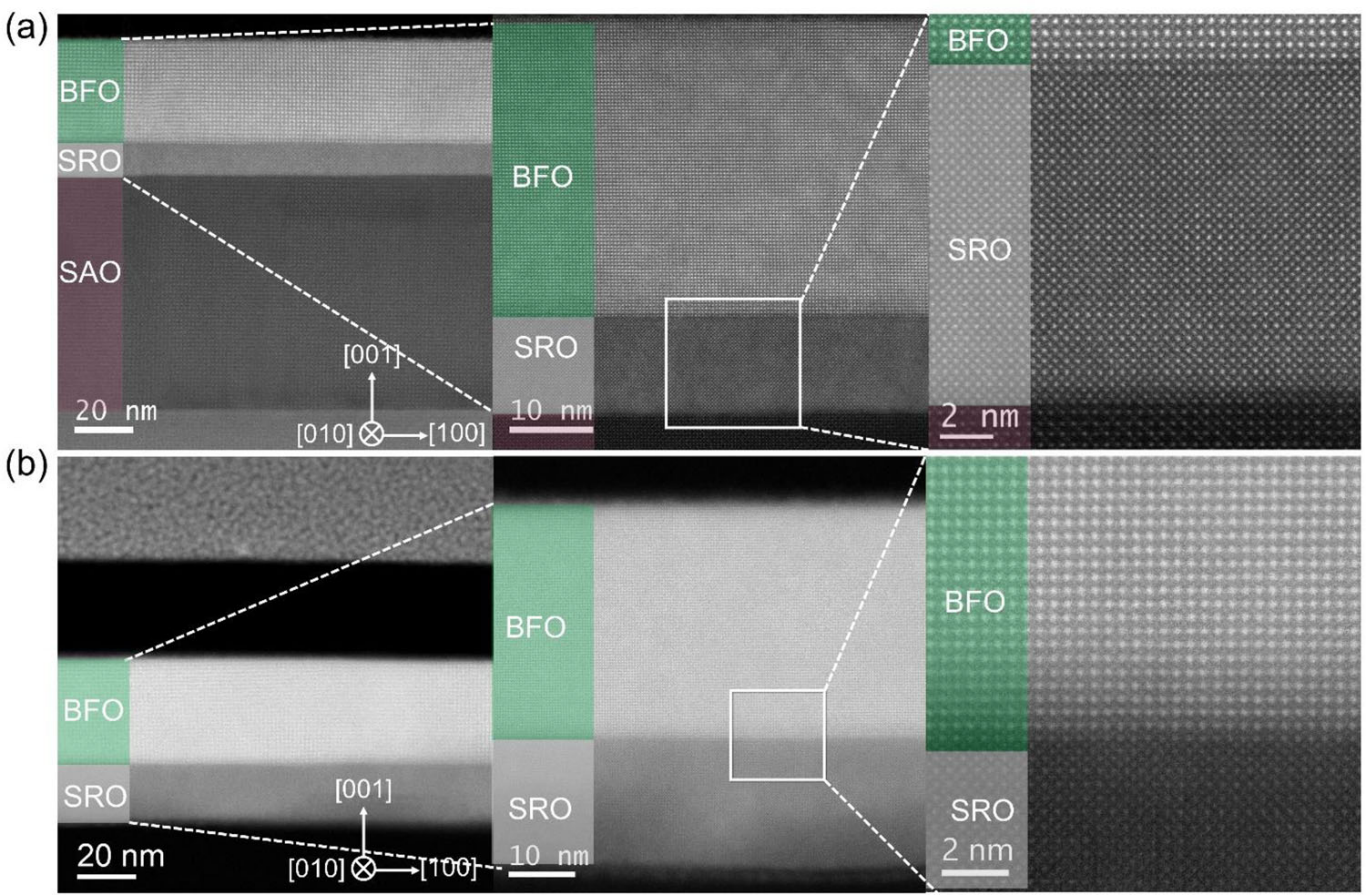
(a) Low‐magnification and atomic‐resolution cross‐sectional HAADF‐STEM images of the as‐grown BFO/SRO/SAO/STO heterostructure and (b) the BFO/SRO membrane transferred to a Si wafer. Labelling is based on the pseudocubic (pc) lattice description of BFO with *a_pc_* = 3.965 Å and α = 89.4°.

Plan‐view characterization of the BFO/SRO membrane on a holey carbon‐coated TEM copper grid is presented in Figure 5 and 6. Both the HAADF‐STEM images and the electron diffraction (ED) patterns show the high crystalline quality of the transferred BFO/SRO bilayer membranes. In addition, Figure 6 highlights the presence of domain walls, visible as darker contrast, along with occasional diffuse steps. The dipole moment map overlaid on the HAADF‐STEM image reveals that these correspond to tail‐to‐tail 109° domain walls. Details on the fitting of the atomic columns and the calculation of the dipole moment maps can be found in the Experimental Section.

**FIGURE 5:**
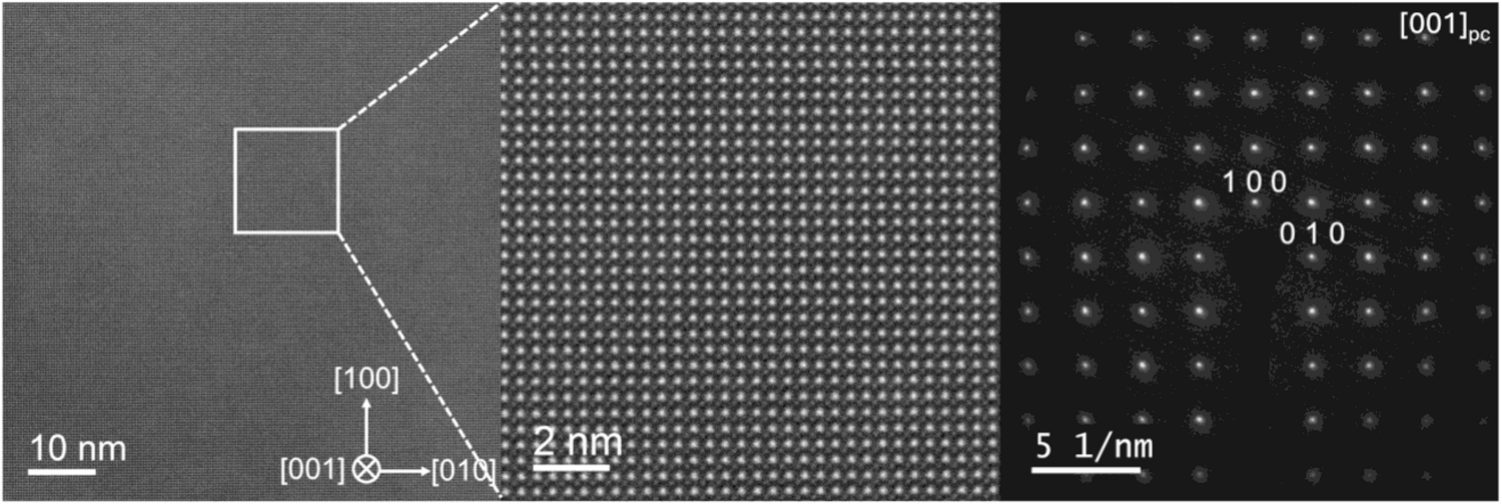
Plan‐view HAADF‐STEM images of a freestanding BFO/SRO membrane on a holey carbon‐coated TEM copper grid and corresponding ED pattern showing the high quality of the transferred membranes. Indexing is based on the pseudocubic (pc) lattice description of BFO.

**FIGURE 6:**
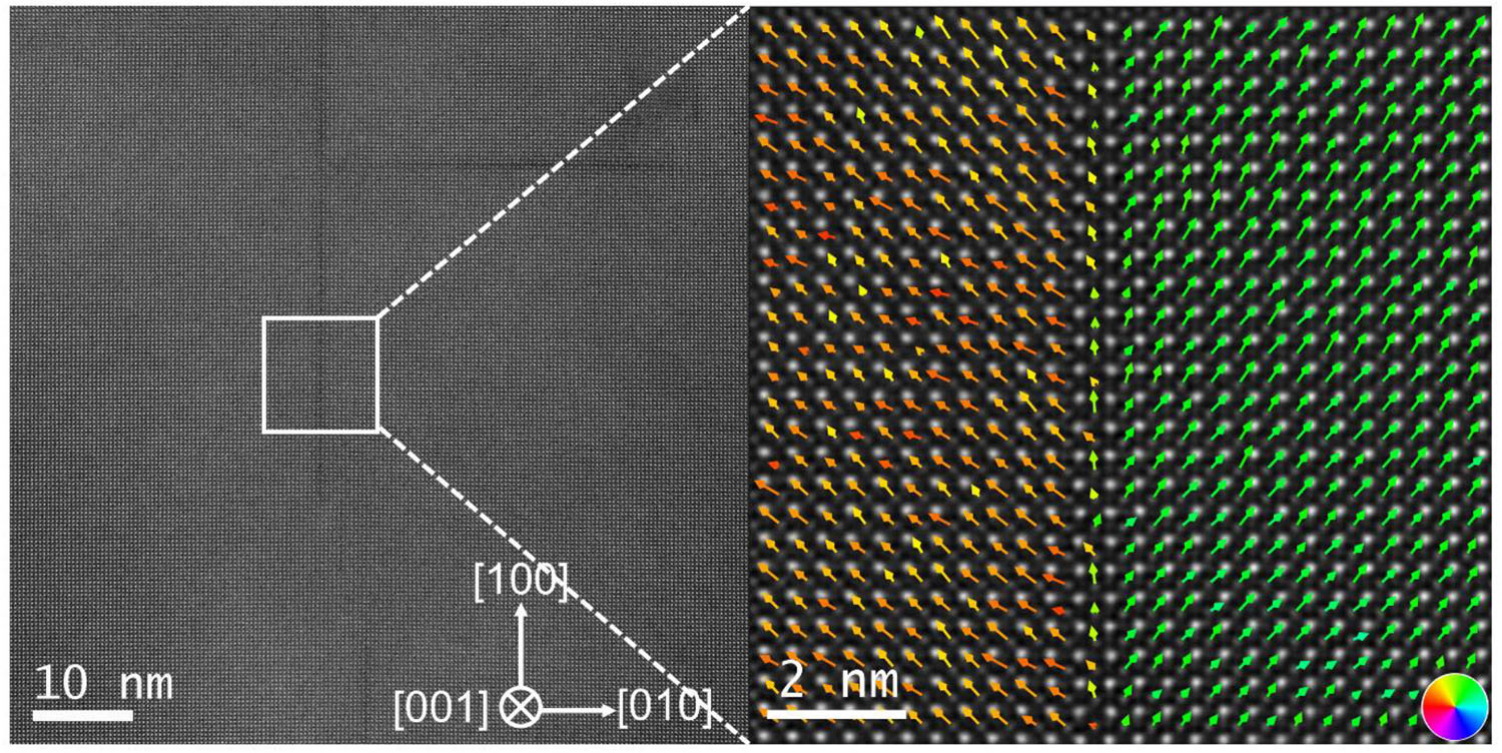
Plan‐view HAADF‐STEM images of a freestanding BFO/SRO membrane showing the presence of tail‐to‐tail 109° domain walls as revealed by the superimposed dipole‐moments map. The arrows show the direction (color wheel) and amplitude (arrow length) of the dipole moments.

Next, we explored the feasibility of fabricating ultrathin BFO membranes with a reduced SRO interlayer to minimize strain at the BFO/SRO interface, a critical factor for probing the intrinsic properties of the BFO membranes. To this end, we grew heterostructures consisting of SRO and BFO layers with thicknesses of 2 and 5 unit cells, respectively. Cross‐sectional and plan‐view HAADF‐STEM images of these ultrathin membranes transferred onto a Si wafer and a holey carbon‐coated TEM copper grid are shown in Figure 7a,b, respectively. These images clearly demonstrate the high crystalline quality of the BFO films. The transferred BFO/SRO bilayer is monocrystalline and exhibits the expected perovskite‐type structure, while the sharp diffraction spots observed in the electron diffraction pattern further confirm the excellent crystallinity of the 5‐unit‐cell freestanding membrane over large areas. An amorphous layer, however, is also clearly visible beneath the crystalline film. The EDX elemental maps in Figure S7 reveal that the amorphous layer is composed of Bi, Al, and Ru oxides. Besides, in the rare and isolated regions where the SRO layer is discontinuous, minor Sr diffusion into the BFO film may also occur. This indicates that a 2‐unit‐cell‐thick SRO layer effectively limits cation diffusion, while slightly thicker layers, ensuring continuity of the buffer, could further suppress diffusion and prevent the formation of an insoluble bottom layer. Although the amorphous layer does not compromise the intrinsic properties of BFO, it presents a challenge for the design of moiré superlattices of stacked BFO membranes, necessitating additional strategies to avoid its formation. Yet, the high crystal quality of the transferred BFO/SRO bilayer allowed us to investigate the structural and ferroelectric properties of the 5‐unit‐cell BFO layer, here using a pulsed laser deposition process. Thus, the ferroelectric state and tetragonal distortion of the ultrathin BFO/SRO bilayer membranes were investigated in cross‐section and are displayed in Figure 8. Figure 8a,b show that the BFO membrane displays an increasing out‐of‐plane polar displacement from bottom to top, while the in‐plane polar displacement remains largely constant. Correspondingly, the *c*/*a* ratio evolves from 0.97 at the interface with SRO, increasing toward the upper surface of the BFO membrane to reach a maximum of 1.05. This value exceeds that of bulk rhombohedral BFO (*c*/*a* = 1.02), marked by the vertical dashed line in Figure 8c for reference. This is fully consistent with the recent report by Ji et al. [10], which demonstrated that single‐crystalline BFO ultrathin membranes undergo a structural phase transition from a rhombohedral‐like to a tetragonal‐like phase below 3 unit cells, accompanied by a substantial increase in polar displacements. In our membranes, the reduced *c*/*a* ratio and the suppressed out‐of‐plane (*δ*y) polar displacements in the first BFO atomic layer above the SRO buffer indicate a residual strain and electrostatic influence from the SRO despite its ultrathin nature. Nevertheless, our results show that ultrathin BFO/SRO membranes grown by pulsed laser deposition exhibit a larger tetragonal distortion than the bulk rhombohedral BFO structure.

**FIGURE 7:**
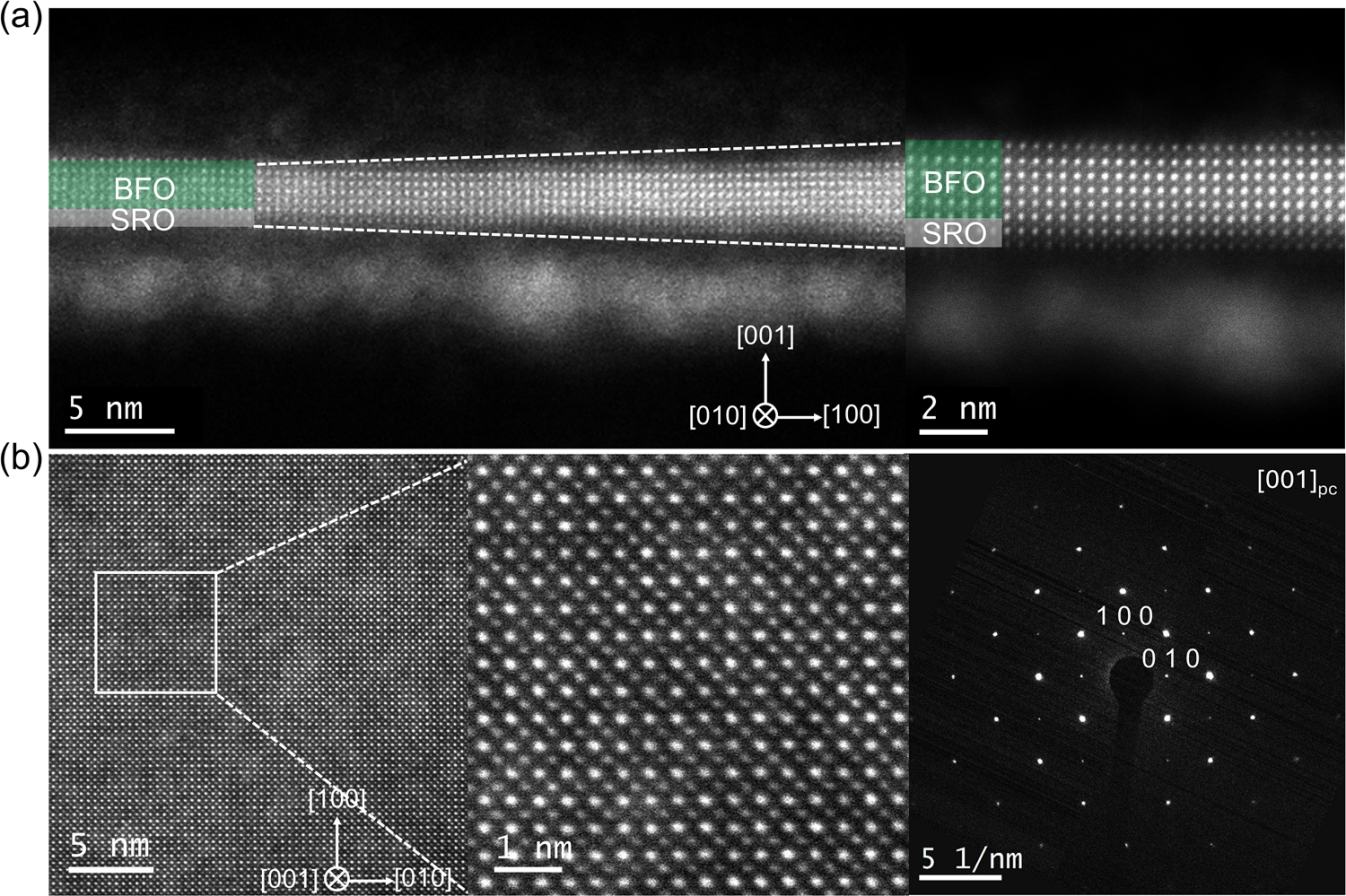
(a) Atomically resolved cross‐sectional HAADF‐STEM images of the ultrathin BFO/SRO bilayer membrane transferred to a Si wafer. (b) Atomically resolved plan‐view HAADF‐STEM images of the freestanding BFO/SRO bilayer membrane transferred to a holey carbon‐coated TEM copper grid and corresponding ED pattern. Indexing is based on the pseudocubic (pc) lattice description of BFO.

**FIGURE 8:**
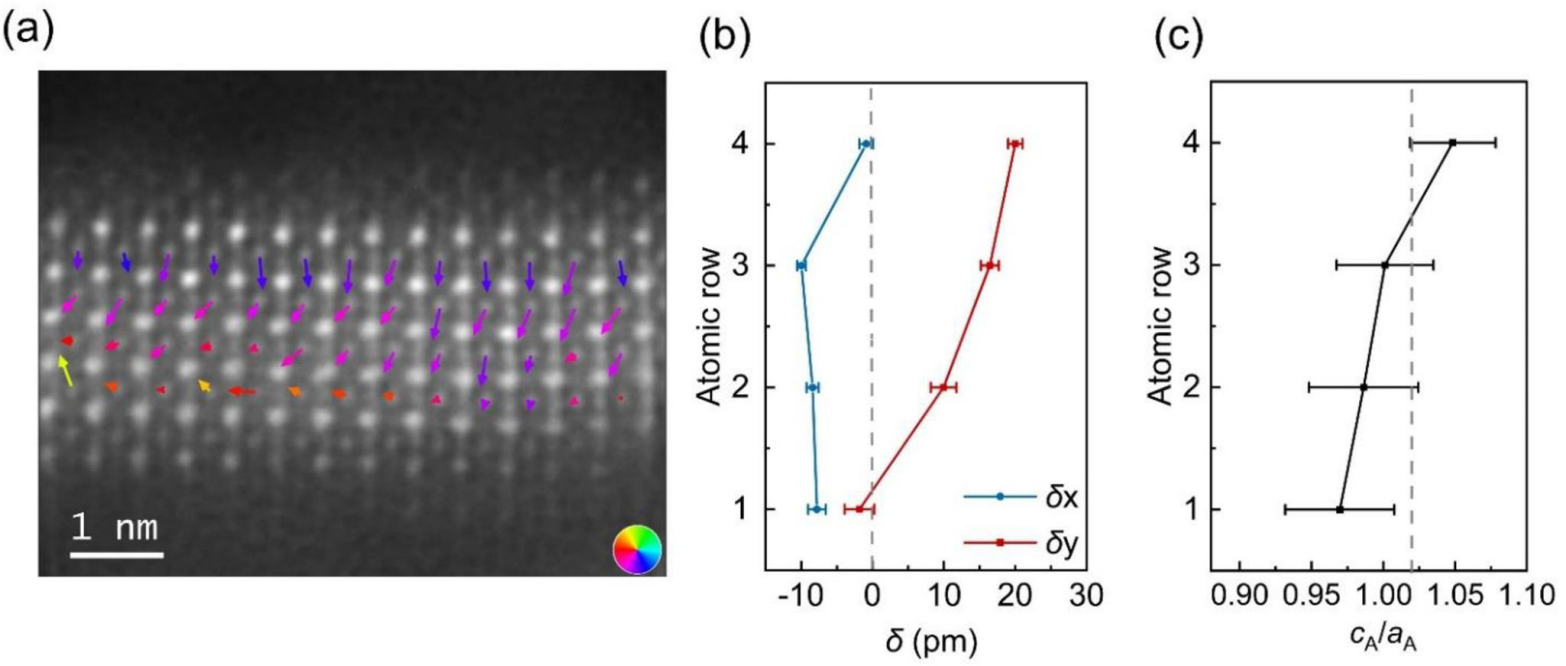
Structural analysis of an ultrathin BFO/SRO bilayer membrane consisting of a 5 unit‐cell BFO layer on a 2 unit‐cell SRO buffer transferred to a Si wafer. (a) Cross‐sectional HAADF‐STEM image with superimposed dipole‐moment map. The polarization vectors are plotted opposite to the displacements of the Fe cations in the image plane from the center of mass of the four nearest Bi neighbors. (b) Corresponding profiles along the [001] direction of the in‐plane (*δ*x) and out‐of‐plane (*δ*y) polar displacements for each atomic row. Error bars indicate the standard deviation. (c) Profile along [001] of the *c/a* tetragonal distortion obtained by peaks‐pair analysis by measuring the distance between pairs of A‐type (A = Bi) atomic columns. Error bars indicate the propagated original uncertainties from the original *a*_A_ and *c*_A_ measurements. The dashed vertical line indicates the *c/a* = 1.02 value of the bulk rhombohedral BFO structure. Note that the two bottom unit cells of the membrane correspond to the SRO layer and their distortions are not mapped. Besides, the ferroelectric distortions of the top BFO layer are also not plotted, as the low intensity of the atomic columns results in poor measurement accuracy.

## 3 Conclusion

In summary, ultrathin freestanding BFO membranes with thicknesses down to 5 unit cells are successfully fabricated by pulsed laser deposition, as confirmed by HAADF‐STEM and SHG. This is achieved by inserting a 2‐unit‐cell SRO layer between the water‐soluble SAO sacrificial layer and the BFO film, effectively suppressing strontium and bismuth diffusion during the high‐energy pulsed laser deposition.

Atomic‐resolution HAADF‐STEM imaging of the ultrathin BFO/SRO membranes transferred onto Si wafers reveals that the BFO films exhibit a polar tetragonal distortion that increases from the SRO interface toward the BFO surface, reaching a consistent *c*/*a* value of 1.05. The corresponding off‐center displacement of the Fe cations relative to the neighboring Bi cations is predominantly out‐of‐plane, with a maximum *δ*y of ∼20 pm. Both the progressive evolution of tetragonal distortion and polar displacements demonstrate that even a 2‐unit‐cell SRO interlayer exerts a substantial clamping effect on the BFO film, underscoring the critical role of interface engineering in ultrathin ferroelectric membranes.

These results establish that freestanding ferroelectric BFO membranes can be reliably fabricated by pulsed laser deposition using SAO as a sacrificial layer, with an SRO interlayer ensuring chemical stability. In stark contrast, direct deposition of BFO on SAO under identical conditions triggers the spontaneous formation of an Aurivillius‐like layered phase devoid of net polarization. Beyond advancing the fabrication of ultrathin ferroelectric membranes, our findings open new opportunities for engineering highly anisotropic layered compounds via PLD using simple perovskite‐based materials, as exemplified here by BFO.

## 4 Experimental Section

### 4.1 Sample Growth and Fabrication

Sr_3_Al_2_O_6_ (SAO) sacrificial layers were grown on SrTiO_3_ (001) substrates using pulsed laser deposition (PLD) and a 248 nm KrF excimer laser. The process was conducted under an oxygen partial pressure of 5 × 10^−5^ mbar and at a substrate temperature of 750°C. Using a BiFeO_3_ (BFO) target, Bi_10_Sr_15_Fe_10_O_46_ (BSFO) Aurivillius films were grown directly on SAO under an oxygen partial pressure of 1.2 × 10^−1^ mbar and at *T*_substrate_ = 710°C. Monocrystalline BFO films were grown under the same conditions used for BSFO with the aid of SrRuO_3_ (SRO) buffer layers with different thicknesses deposited under an oxygen partial pressure of 1.2 × 10^−1^ mbar and *T*_substrate_ = 700°C. To transfer the membranes for plan‐view investigations, the as‐grown films together with the substrate were immersed in deionized water at room temperature. Once the SAO was etched, the substrate, which still had the film on it, was carefully removed from the water and slowly submerged back into the water. This facilitates the detachment of the films from the STO substrates, resulting in large‐area freestanding membranes floating on the water surface. A holey carbon‐coated TEM copper grid was then used to scoop up the membranes. The transfer of the membranes onto Si substrates for cross‐sectional investigations was carried out with the aid of PDMS. First, the PDMS was placed onto the film surface and both the sample and the PDMS were immersed in deionized water until the SAO layer dissolved and the film/PDMS detached from the substrate. The film/PDMS was then applied to a Si substrate and heated at 80°C for 60 min. Finally, the PDMS was peeled off, leaving the freestanding membrane on the new substrate. For growth, 5 × 5 × 0.5 mm^3^ STO substrates were cut into four pieces, yielding membranes of roughly 2 × 2 mm^2^.

### 4.2 Optical Secondary Harmonic Generation (SHG)

The SHG measurements were performed with a reflection geometry under a 45° incidence angle. During the polarizer measurement, the linear polarization of the incoming fundamental light is rotated while the generated SHG signal is probed with a fixed *s*‐polarized configuration. The fundamental probe beam was generated by an amplified Ti:Sapphire laser system with a pulse duration of 50 fs at the repetition rate of 1 kHz. Its wavelength was then converted from 800 to 1200 nm using an optical parametric amplifier. The pulse energy of the probe beam was set to 10 µJ and subsequently focused onto the sample with a probe diameter of 250 µm. The SHG signal generated by the sample at 600 nm was selected by a monochromator and collected by a photomultiplier tube, which was then converted into an electrical signal. The polarizer measurement simulation is conducted using the open‐source *#*SHAARP package [32].

### 4.3 X‐Ray Diffraction

X‐ray symmetric θ−2θ scans were performed using a Panalytical X'Pert3 MRD four‐circle diffractometer using Cu K_α1_ x‐ray radiation at a wavelength of 1.5406 Å.

### 4.4 Transmission Electron Microscopy (TEM)

Cross‐sectional electron transparent samples for TEM investigations were prepared using a FEI Helios 660 G3 UC focused ion beam (FIB) operated at acceleration voltages of 30 and 5 kV after deposition of C and Pt protective layers. Scanning electron microscopy (SEM) images were acquired using the same instrument. Electron diffraction (ED) was performed using a JEOL 2200FS TEM/STEM microscope operated at 200 kV. High‐angle annular dark‐field scanning TEM (HAADF‐STEM) imaging and energy‐dispersive x‐ray (EDX) spectroscopy were carried out using a probe‐corrected FEI Titan Themis microscope equipped with ChemiSTEM technology. The microscope was operated at an accelerating voltage of 300 kV, probe convergence semi‐angle of 18 mrad and collecting semi‐angles of 66–200 mrad. The HAADF‐STEM images for dipole‐moment mapping were obtained from averaging time series consisting of 14 frames (2048 × 2048, 1 µs) by nonrigid registration using the SmartAlign software [33]. Then, the atomic column fitting was performed on the averaged HAADF‐STEM images using the Python library Atomap [34] after applying a Python‐based probe deconvolution algorithm. The polarization maps were derived from the relative displacements of the two cation sublattices present in the BFO perovskite‐type structure. Thus, the polar displacement of the Fe position on the image plane from the center of mass of the four nearest Bi neighbors was determined. Since the Fe cations are displaced along with the O octahedra, the dipole moments are represented here in the opposite direction to the displacement of the Fe cations. The tetragonal distortions of the membranes were analyzed by peak‐pair analysis by measuring the distance between pairs of Bi‐atomic columns along the in‐plane and out‐of‐plane directions. Thus, the interatomic distances of the *A*‐sublattice are defined as *a*_A_ and *c*_A_ and are parallel to [100] and [001], respectively. Subsequently, the *c*_A_/*a*_A_ ratio is extracted for each atomic row.

## Conflicts of Interest

The authors declare no conflicts of interest.

## Supporting information




**Supporting File**: smll74989‐sup‐0001‐SuppMat.docx.

## Data Availability

The data that support the findings of this study are available from the corresponding author upon reasonable request.
