# Supplementary material for: Ultrathin Freestanding BiFeO3 Membranes Grown by Pulsed Laser Deposition
Source: Small. 2026 Aug 18;22(56):e11535. doi: 10.1002/smll.202511535 (PMC13653344; doi:10.1002/smll.202511535)
Supplement: Supplementary file 1 [file SMLL-22-e11535-s001.docx]

## Supporting Information

## Ultrathin freestanding BiFeO_3_ membranes grown by pulsed laser deposition

Yifan Zhao,^1,*^ Bixin Yan,^2,*^ Christoph Dräyer,^2^ Morgan Trassin,^2^ Marta D. Rossell^1^

^1^ Electron Microscopy Center, Empa, Swiss Federal Laboratories for Materials Science and Technology, 8600 Dübendorf, Switzerland

^2^ Department of Materials, ETHZ, 8093 Zürich, Switzerland

* These authors contributed equally to the work.

I. **Transferred Aurivillius-like Bi_10_Sr_15_Fe_10_O_46_ (BSFO) membranes**

The Aurivillius-like BSFO films were detached from the STO substrates by soaking them in deionized water and subsequently transferred to a Si wafer with the aid of PDMS. Complete film detachment takes 20 to 30 minutes under full immersion when collecting the film on a carbon-coated TEM copper grid; however, this process increases to 1–2 hours when a PDMS support layer is utilized for the BSFO transfer. Cross-sectional, electron-transparent lamellae for imaging were prepared via focused ion beam (FIB) milling. Figure S1a displays the cross-sectional HAADF-STEM images of a transferred BSFO membrane on Si, with the corresponding EDX elemental maps presented in Figure S1b. The membrane comprises a crystalline upper region and an amorphous lower region, each measuring approximately 10 nm in thickness. Notably, the thickness of this amorphous layer corresponds closely to the pre-dissolution interfacial region of the SAO layer, which exhibited a reduced Sr:Al ratio of 54:46 within the topmost 10–20 nm zone compared to approximately 57:43 in the underlying bulk sacrificial film. The EDX maps reveal that Sr diffuses within the Aurivillius membrane and preferentially accumulates in the perovskite blocks. Concurrently, the amorphous layer is composed of Bi and Fe oxides, suggesting that Bi and Fe interdiffuse into the SAO sacrificial layer to form an insoluble amorphous phase. This interdiffusion explains the absence of Bi in the perovskite-type blocks of the BSFO Aurivillius-like phase. Additionally, trace amounts of Al are detected around the membrane, originating from the dissolved SAO sacrificial layer.

The BSFO films were also transferred to carbon-coated TEM grids for plan-view investigations. Figure S1c shows plan-view HAADF-STEM images of an Aurivillius-like membrane and the corresponding electron diffraction (ED) pattern demonstrating the high crystalline quality of the transferred membranes. Note that in the atomically resolved HAADF-STEM image, it is not possible to distinguish between the *A*- and *B*-cationic sublattices of the *AB*O_3_ perovskite structure because in the Aurivillius-like phases neighboring perovskite intergrowths are displaced by half a unit cell and the *A*- and *B*-atomic columns of the different perovskite-type SrFeO_3_ intergrowths overlap along the out-of-plane [100] direction. Further, a close inspection of the ED pattern in Figure S1c reveals a diffuse ring corresponding to the presence of the amorphous region below the transferred BSFO membrane, which is clearly visible in the cross-sectional HAADF-STEM image in Figure S1a.

SEM images of the BSFO membrane transferred onto a Si substrate are shown in Figure S2. The released membrane shows a high density of defects, mainly cracks and surface contamination. The cracks are attributed to the amorphous layer beneath the crystalline Aurivillius film (see Figure S1a,b), which likely modifies the mechanical properties and decreases elastic compliance. The contamination likely stems from chemical modification of the SAO layer during PLD growth of the overlying film, affecting its dissolution behavior and resulting in incomplete removal during wet etching.

**Figure S1.** a) Low magnification and atomic resolution cross-sectional HAADF-STEM images of the BSFO Aurivillius-like membrane transferred onto a Si substrate imaged along the [015]_o_ direction of the BSFO phase. b) Corresponding EDX elemental maps. The EDX maps for Bi, Sr, Al, Fe and O were obtained by integrating the Bi−L, Sr-K, Al-K, Fe-K and O-K edge intensities, respectively. c) Atomically resolved plan-view HAADF-STEM images of a freestanding BSFO membrane transferred on a holey carbon-coated TEM copper grid and corresponding ED pattern. Indexing is based on the BSFO orthorhombic (o) *C*222 space group.

**Figure S2.** SEM images of the BSFO Aurivillius-like membrane transferred onto a Si substrate showing the presence of cracks and surface contamination. The white arrow marks the membrane edge.

II. **As-grown BFO/SRO/SAO/STO heterostructure**

**Figure S3.** SHG polarizer measurement from the as-grown BFO/SRO/SAO/STO heterostructure and simulation of the BFO layer with point group *m* by *#*SHAARP in reflection geometry with a 45° incidence angle for fixed *s*-polarized SHG light. The experimentally measured data are plotted with orange dots, while the simulation is plotted as a solid black line.

**Figure S4.** Reciprocal space mapping of the BFO/SRO/SAO/STO heterostructure. The SAO and BFO peaks exhibit the same Q∥value, which is smaller than that of the STO substrate, indicating a shared in-plane lattice parameter and relaxation with respect to STO.

**Figure S5.** a) Low magnification HAADF-STEM image and corresponding EDX elemental maps of the as-grown BFO/SRO/SAO/STO heterostructure. The EDX maps for Bi, Sr, Al, Fe, Ru, Ti and O were obtained by integrating the Bi−L, Sr-K, Al-K, Fe-K, Ru-K, Ti-K and O-K edge intensities, respectively.

III. **Transferred BFO/SRO bilayer membranes**

**Figure S6.** SEM images of a BFO/SRO membrane transferred onto a Si substrate. The membrane consists of a bilayer comprising a 20 nm thick BFO film on a 15-unit-cell SRO buffer. The white arrow indicates the membrane edge. The released membrane is crack-free over several hundred μm².

**Figure S7.** a) Low magnification HAADF-STEM image and corresponding EDX elemental maps of the ultrathin BFO/SRO bilayer membrane transferred onto a Si wafer, acquired in an uncommon region exhibiting discontinuity of the SRO layer. The BiFeO₃ layer is clearly visible at the top, followed by a crystalline SrFeOₓ layer and an underlying amorphous layer composed of Bi, Ru, Al, and O. The EDX maps for Bi, Sr, Fe, Ru, Al and O were obtained by integrating the Bi−L, Sr-K, Al-K, Fe-K, Ru-K and O-K edge intensities, respectively.
